# Inflammatory and metabolic markers in relation to outcome of in vitro fertilization in a cohort of predominantly overweight and obese women

**DOI:** 10.1038/s41598-022-17612-2

**Published:** 2022-08-03

**Authors:** Henrik Svensson, Snorri Einarsson, Daniel Olausson, Linda Kluge, Christina Bergh, Staffan Edén, Malin Lönn, Ann Thurin-Kjellberg

**Affiliations:** 1 Department of Laboratory Medicine, Institute of Biomedicine, Sahlgrenska Academy, University of Gothenburg, 405 30 Gothenburg, Sweden; 2 Department of Obstetrics and Gynecology, Institute of Clinical Sciences, Sahlgrenska Academy, University of Gothenburg, 405 30 Gothenburg, Sweden; 3 Livio Reykjavik, Álfheimar 74, 104 Reykjavík, Iceland; 4 Department of Clinical Chemistry, Region Västra Götaland, Sahlgrenska University Hospital, Bruna stråket 16, 413 45 Gothenburg, Sweden; 5 Department of Reproductive Medicine, Region Västra Götaland, Sahlgrenska University Hospital, Blå stråket 6, 413 45 Gothenburg, Sweden; 6 Department of Internal Medicine, Institute of Medicine, Sahlgrenska Academy, University of Gothenburg, 405 30 Gothenburg, Sweden

**Keywords:** Medical research, Inflammation, Endocrinology, Endocrine system and metabolic diseases, Endocrine system and metabolic diseases, Reproductive disorders, Reproductive signs and symptoms

## Abstract

For overweight and obese women undergoing in vitro fertilization (IVF) the pregnancy and live birth rates are compromised while the underlying mechanisms and predictors are unclear. The aim was to explore the association between adipose tissue-related inflammatory and metabolic markers and the pregnancy and live birth outcome of IVF in a cohort of predominantly overweight and obese women. Serum samples, fulfilling standardizing criteria, were identified from 195 women having participated in either the control (n = 131) or intervention (n = 64) group of a randomized controlled trial (RCT), seeking to evaluate the effect of a weight reduction intervention on IVF outcome in obese women. Serum high-sensitivity C-reactive protein (hsCRP) and the adipokines leptin and adipocyte fatty acid-binding protein (AFABP) were analyzed for the whole cohort (n = 195) in samples collected shortly before IVF [at randomization (control group), after intervention (intervention group)]. Information on age, anthropometry [BMI, waist circumference, waist-to-height ratio (WHtR)], pregnancy and live birth rates after IVF, as well as the spontaneous pregnancy rate, was extracted or calculated from collected data. The women of the original intervention group were also characterized at randomization regarding all variables. Eight women [n = 3 original control group (2.3%), n = 5 original intervention group (7.8%)] conceived spontaneously before starting IVF. BMI category proportions in the cohort undergoing IVF (n = 187) were 1.6/20.1/78.3% (normal weight/overweight/obese). The pregnancy and live birth rates after IVF for the cohort were 35.8% (n = 67) and 24.6% (n = 46), respectively. Multivariable logistic regression revealed that none of the variables (age, hsCRP, leptin, AFABP, BMI, waist circumference, WHtR) were predictive factors of pregnancy or live birth after IVF. Women of the original intervention group displayed reductions in hsCRP, leptin, and anthropometric variables after intervention while AFABP was unchanged. In this cohort of predominantly overweight and obese women undergoing IVF, neither low-grade inflammation, in terms of hsCRP, other circulating inflammatory and metabolic markers released from adipose tissue (leptin, AFABP), nor anthropometric measures of adiposity or adipose tissue distribution (BMI, waist, WHtR) were identified as predictive factors of pregnancy or live birth rate.

*Trial registration*: ClinicalTrials.gov number, NCT01566929. Trial registration date 30-03-2012, retrospectively registered.

## Introduction

The global prevalence of obesity has increased substantially over the last decades. In women of reproductive age, overweight and obesity negatively affects fertility^1,2^ and obstetric outcome with an elevated risk of miscarriage^3,4^ and obstetric and neonatal complications^5–7^. Overweight and obesity also seem to negatively affect outcome of in vitro fertilization (IVF) suggesting that the underlying mechanism extends beyond an ovulatory disorder. Observational studies and meta-analyses indicate that overweight and obese women undergoing IVF have lower pregnancy and live birth rates compared with normal weight women^8–14^. However, in a large randomized trial in obese women, no increase in live birth rate was observed after considerable weight loss compared with women not losing weight^15^. It should be noted though that the study was not powered to detect a small increase in live births due to weight reduction. Taken together, the value of weight reduction recommendations to obese women scheduled for IVF needs further investigation, and the underlying factors linking overweight and obesity to poor IVF outcome should be evaluated.

Adipose tissue is a dynamic endocrine organ involved in multiple processes including glucose homeostasis, steroid production, inflammation and reproduction^16,17^. The pleiotropic role of adipose tissue is mainly based on its ability to synthesize and release a large number of e.g. hormones, cytokines, acute-phase proteins and growth factors, collectively termed adipokines^18,19^. The adipokines can act locally and as signaling molecules in other organs. Therefore, the secretion of adipokines from adipose tissue may underlie many components of obesity-related disorders. In the obese state, the release of pro-inflammatory and immune-related molecules from adipose tissue is induced, resulting in a state of low-grade, chronic inflammation associated with alterations in immune cell populations^20^. Systemic low-grade inflammation, as indicated by high high-sensitivity C-reactive protein (hsCRP) levels, has been associated with reduced fecundability, but not independently from adiposity^21^. Further, excess release of free fatty acids (FFA) and differential secretion of adipokines from obese adipose tissue may influence the hypothalamic–pituitary–gonadal (HPG) axis and the endometrium negatively thus affecting fertility and IVF outcomes^22–24^.

It is established that there is considerable heterogeneity in the degree of metabolic and inflammatory characteristics associated with obesity^25,26^. The distribution of adipose tissue, the adipose tissue cellular composition, and the adipocyte size are important factors in this context^18,19,25–27^. Interestingly, it has been reported that a lower-body fat distribution in women is being more favorable than a central distribution, not only from a cardio-metabolic perspective but when it comes to fecundity and pregnancy and live birth rates after assisted reproductive technology^28–30^.

The aim of the present study was to identify factors linking overweight and obesity to IVF outcome. Low-grade inflammation, in terms of hsCRP, other circulating inflammatory and metabolic markers released from adipose tissue [leptin, adipocyte fatty acid-binding protein (AFABP)], and anthropometric measures of adiposity and adipose tissue distribution [BMI, waist circumference, waist-to-height ratio (WHtR)] were analyzed and evaluated as predictors of pregnancy and live birth rate in a cohort of predominantly overweight and obese women scheduled for IVF.

## Material and methods

### Subjects and study design

A prospective, multicenter RCT evaluating the effect of an intensive weight reduction program on live birth rates in obese women (BMI ≥ 30 and < 35 kg/m^2^) scheduled for IVF was performed between 2010 and 2016 in the Nordic countries^15^. In brief, participating women were randomized and examined, including blood sampling and anthropometric measurements, at a first study visit. Women randomized to the control group received IVF treatment as soon as possible after the first study visit with no previous intervention. Women randomized to the intervention group were assigned a low calorie diet (LCD) for 12 weeks, a following weight maintenance period of 2–5 weeks, and a repeated examination according to the protocol previously used at a second study visit followed by IVF treatment^15^. The LCD was a liquid formula diet with a daily energy intake of 880 kcal (Modifast, Nutrition & Santé, France). During the LCD period, all women had scheduled visits with a health professional, dietician or nurse, at weeks 0, 2, 5, 8, and 12 where weight was recorded. After termination of the 12-week LCD period, the women were scheduled for individual visits with a dietician during the weight maintenance period for the re-introduction of solid foods. Women unable to complete the LCD treatment received individualized weight loss counselling until the start of IVF treatment. The women started IVF after the weight intervention period regardless of to what extent weight reduction was achieved^15^.

**Figure 1:**
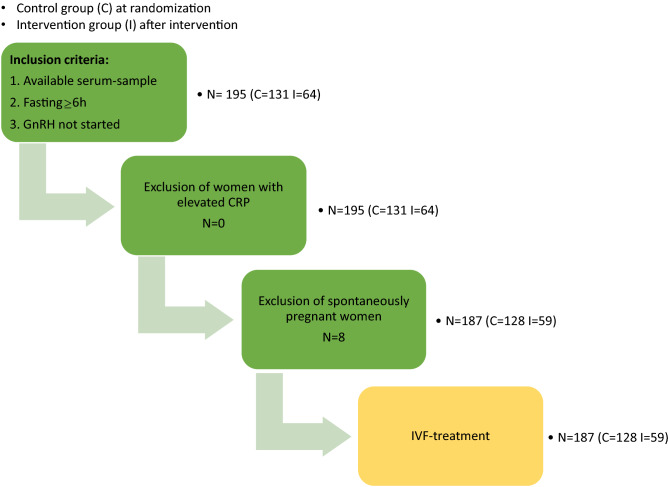
Flowchart of study design.

In the present study, 195 women from the original RCT^15^ were included, all having serum samples stored shortly before IVF, i.e. at randomization for the original control group (n = 131) and after intervention for the original intervention group (n = 64). All included samples were collected according to standardizing criteria i.e. after the woman had been fasting for at least 6 h and before downregulation with gonadotropin releasing hormone (GnRH)-agonist (Fig. 1). Corresponding samples, collected at randomization for the original intervention group, were also identified. All samples were analyzed regarding hsCRP, leptin and adipocyte fatty acid-binding protein (AFABP).

Information on age, anthropometry, pregnancy and live birth rates after IVF, the spontaneous pregnancy rate after the examination closest to IVF, basal blood chemistry, sex steroids and sex hormone binding globulin (SHBG), smoking habits, and ± PCOS was extracted or calculated from collected data.

All participants provided oral and written informed consent. The trial was approved by research ethics committees in Sweden, Denmark and Iceland. All experiments were performed in accordance with guidelines supplied by the manufacturers and relevant regulations.

### Anthropometry and biochemical assays

Body height, body weight, waist circumference, BMI and waist-to-height ratio were measured or calculated by standard protocols. Blood sampling was performed according to standard clinical procedures. Serum samples were stored at − 80 C until analysis. ELISA was used to measure leptin (Quantikine Human Leptin ELISA kit, DLP00, R&D Systems, Minneapolis, MN; interassay coefficient of variation (CV) 3.6% at 4.0 ng/ml), AFABP (Quantikine Human FABP4 ELISA kit, DFBP40, R&D Systems; interassay CV 5.3% at 12.6 ng/ml), and hsCRP (Quantikine Human C-reactive Protein ELISA kit, DCRP00, R&D Systems; interassay CV 3.1% at 3.2 mg/L). All assays for basal blood chemistry were performed in fresh samples at each university hospital according to Clinical Chemistry Laboratory routines.

### Statistical analysis

Results are expressed as mean ± SD (continuous variables) or number and percentage (categorical variables). Women with CRP values defined as outliers (> 75th percentile + 3 × inter-quartile range; > 17.1 mg/L) were evaluated in detail in order to identify and exclude any woman having an acute inflammation. Multiple logistic regression, applying full models and backward stepwise selection, with *p* < 0.05 as condition in each step for staying in the model, was used to identify factors independently associated with IVF outcomes (pregnancy and live birth, respectively). Candidate variables were age, hsCRP, leptin, AFABP, BMI and waist circumference. Alternative analyses were also performed where waist circumference was substituted by WHtR. The linearity assumption for continuous variables was checked by calculating the difference in − 2 log likelihood between the models with and without the variable in question squared included. Logistic regression was also used to analyze possible interactions between randomization group and candidate variables regarding outcome. Spearman’s rank correlation was used to evaluate associations between variables and between changes in leptin and AFABP during intervention. Wilcoxon’s signed rank test was used for testing the effect of the intervention on candidate variables within the original intervention group. All tests are two-sided and *p* values below 0.05 were considered statistically significant. All analyses was performed using SPSS version 25 for MacOS (SPSS, Chicago, IL).

The original power calculation, made prior to the RCT^15^, was based on unpublished raw data of a previous study^31^. In brief, the live birth rate was 12.5% for obese women and 26.3% for women with a normal weight. To find a difference of 13%, 152 patients were needed in each group, giving a total of 304 patients (significance 5%, power 80%). To compensate for dropouts, the sample size was increased to 316. No loss of follow up was expected^15^. The current exploratory post-hoc analysis was based on the blood samples collected during the RCT and which met the standardizing criteria.

### Ethic approval and consent to participate

All participants provided oral and written informed consent. The trial was approved by research ethics committees in Sweden [Regionala etikprövningsnämnden i Göteborg (Dnr 292-10)], Denmark [De videnskabsetiske komiteer for region Huvudstad (H-2-2012-127)] and Iceland [Visindasidanend (13-139-S1)].

## Results

Out of the 195 women included in the present study, based on available serum samples fulfilling standardizing criteria, no woman was excluded due to acute inflammation as indicated by increased hsCRP (Fig. 1). One woman displayed hsCRP above 17.1 mg/L (original intervention group; 18.6 mg/L at randomization, 14.5 mg/L shortly before IVF) but her overall status did not indicate acute inflammation (BMI 34.0 kg/m^2^ at randomization, 34.1 kg/m^2^ shortly before IVF; leptin 27 ng/mL at randomization, 23 ng/mL shortly before IVF).

Eight women [n = 3 original control group (2.3%), n = 5 original intervention group (7.8%)] conceived spontaneously after the examination closest to IVF (Fig. 1). The remaining 187 women underwent IVF.

### Characteristics of the cohort undergoing IVF

Characteristics of the cohort undergoing IVF (n = 187) are presented in Table 1. BMI category proportions were 1.6/20.1/78.3% (normal weight/overweight/obese). Mean BMI was 31.7 (range 24.2–35.1) kg/m^2^. Three women were normal-weight (BMI 24.2, 24.5, 24.7 kg/m^2^). The analyzed serum biomarkers displayed more than tenfold (AFABP), 25-fold (leptin), and 150-fold (hsCRP) variability, respectively. Information on basal blood chemistry, sex steroids and sex hormone binding globulin (SHBG), PCOS, and smoking habits are presented in Supplemental Table 1.

**Table 1:** Characteristics of the cohort at the examination closest to IVF (n = 187).

	Mean ± SD	Range
Age	31.7 ± 4.14	22.3–38.0
Height (cm)	165.8 ± 6.55	146.0–182.0
Weight (kg)	87.3 ± 10.00	66.0–114.7
BMI (kg/m^2^)	31.7 ± 2.71	24.2–35.10
Waist (cm)	97.2 ± 9.21	74.0–123.0
Waist-to-height ratio	0.59 ± 0.06	0.41–0.74
AFABP (ng/mL)	23.2 ± 10.86	6.7–86.1
Leptin (ng/mL)	28.5 ± 14.44	3.7–97.0
hsCRP (mg/L)	3.8 ± 3.30	0.1–16.3

Spearman rank correlation revealed positive correlations between many of the studied variables (*p* < 0.01–0.05) (Table 2). HsCRP was correlated with all other studied variables, except weight.

**Table 2:** Correlations between studied variables at the examination closest to IVF (n = 187).

	AFABP	Leptin	hsCRP
AFABP	1	0.129	0.274**
Leptin	0.129	1	0.162*
hsCRP	0.274**	0.162*	1
Weight	0.225**	0.425**	0.140
BMI	0.199**	0.493**	0.297**
Waist	0.135	0.362**	0.239**
Waist-to-height ratio	0.108	0.345**	0.311**

Values are Spearman rank correlation, ρ.

*p < 0.05; **p < 0.01.

The pregnancy and live birth rates in the cohort after IVF were 35.8% (n = 67/187) and 24.6% (n = 46/187), respectively.

### Multivariable regression

The linearity assumption was fulfilled for all variables and none of them was identified as a significant predictor of pregnancy or live birth after IVF using full models (Table 3) or backward stepwise selection. Neither was any interaction between treatment group and variables tested found.

**Table 3:** Multivariable logistic regression analyses.

	Live birth	Pregnancy
Age	− 0.048; 0.044; 0.274; 0.953	− 0.037; 0.039; 0.336; 0.964
AFABP (ng/ml)	0.001; 0.017; 0.962; 1.001	− 0.005; 0–015; 0.745; 0.995
Leptin (ng/ml)	− 0.001; 0.015; 0.970; 0.999	− 0.001; 0.013; 0.964; 0.999
hsCRP (mg/l)	− 0.090; 0.064; 0.161; 0.914	0.031; 0.049; 0.535; 1.031
BMI (kg/m^2^)	− 0.054; 0.094; 0.565; 0.947	− 0.104; 0.084; 0.214; 0.901
Waist (cm)	0.025; 0.025; 0.331; 1.025	0.026; 0.022; 0.253; 1.026
Constant	− 0.032; 2.623; 0.990; 0.968	1.365; 2.354; 0.562; 3.916
Waist-to-height ratio	2.204; 4.297; 0.608; 9.062	− 0.430; 3.777; 0.909; 0.651

Multivariate logistic regressions with live birth or pregnancy as dichotomous dependent variables. Values are: β; SE; P; OR. In a second analysis waist was replaced by waist-to-height ratio.

### Effects of intervention

**Figure 2:**
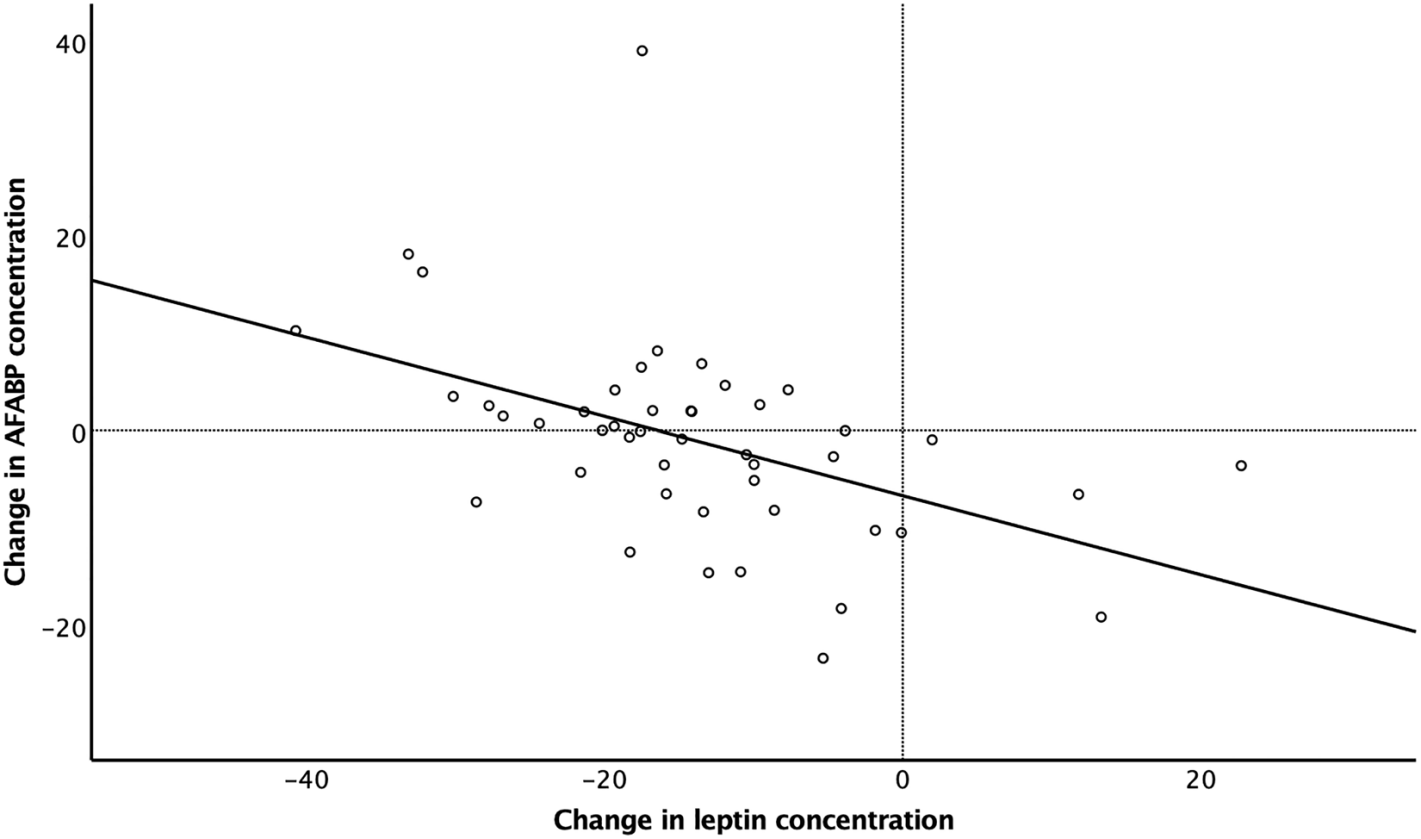
Correlation between change in serum leptin concentration (ng/mL) and change in serum AFABP concentration (ng/mL) during intervention (rho = − 0.530, *p* < 0.001, n = 46).

Women belonging to the original intervention group displayed reductions in hsCRP, leptin, and anthropometric variables after intervention while AFABP was unchanged (Table 4). The weight change was − 10.2 ± 6.99 kg (range − 23.3 to + 7.9), n = 46 (13 women were not fasting at randomization). Delta AFABP was negatively correlated with delta leptin (rho = − 0.530, *p* < 0.001, n = 46) (Fig. 2).

**Table 4:** Effects of weight loss intervention.

	Randomization (n = 46)	Examination closest to IVF (n = 59)	*p***°**
Weight (kg)	92.7 ± 7.12	79.1 ± 8.32	**< 0.01**
BMI (kg/m^2^)	33.5 ± 1.19	28.6 ± 2.44	**< 0.01**
Waist (cm)	100.9 ± 6.88	89.9 ± 7.99	**< 0.01**
Waist-to-height ratio	0.61 ± 0.04	0.54 ± 0.05	**< 0.01**
AFABP (ng/mL)	23.9 ± 10.25	21.8 ± 9.44	0.287
Leptin (ng/mL)	34.3 ± 10.25	20.6 ± 11.98	**< 0.01**
hsCRP (mg/L)	4.5 ± 4.51	3.1 ± 3.56	**< 0.01**

Significant values are in [bold].

Values are mean ± SD or percentage.

°Related-Samples Wilcoxon Signed Rank test, intervention group at randomization versus examination closest to IVF.

## Discussion

In this study, neither hsCRP, a key indicator of low-grade inflammation, other inflammatory and metabolic markers released from adipose tissue in terms of leptin and AFABP, nor measures of adiposity or adipose tissue distribution, i.e. BMI, waist and WHtR, were identified as predictors of pregnancy or live birth rate after IVF in a cohort of women with a wide range of BMI indices.

Thus, the negative influence of high BMI when it comes to pregnancy and live birth rates after IVF was not confirmed for the present cohort although well established in previous observational studies^8–14^. In a large retrospective cohort study from the USA^13^ the difference in implantation rate between the reference group of women of normal weight and women with overweight on one hand, and women with obesity class I on the other, was 1.2% and 2.6%, respectively, and the live birth rate difference was 1.6% and 3.4%, respectively. These subtle differences in this large data set were shown to be statistically significant but indicate that larger cohorts may be required for statistical power and that the underlying factors, related to adipose tissue, may be hard to detect.

Interestingly, a recent prospective cohort study including 1889 infertile couples undergoing IVF suggests that any negative impact of obesity on IVF outcomes, including live birth rate, may be ameliorated with the use of IVF/intracytoplasmic sperm injection (ICSI) and frozen-warmed embryo transfer. Live birth rates were similar in women of all BMI categories, and the authors concluded that frozen-warmed embryo transfer, with no or less hormonal stimulation, might be more optimal concerning embryo-endometrial synchrony, particularly in the overweight and obese population. Most previous studies, demonstrating a negative effect of obesity on IVF outcome, consisted largely of fresh transfers^32^. Other studies using frozen-warmed embryo transfer have reported similar results^33,34^.

C-reactive protein (CRP) is a general marker of inflammation that is chronically elevated in obesity, reflecting the low-grade inflammatory state of adipose tissue. Circulating concentrations of hsCRP is positively correlated to BMI^35^. A recent systematic review on CRP and assisted reproductive technique (ART) outcomes highlights a possible detrimental impact of preconception high circulating CRP concentration on ART outcomes, but concludes that the predictive value of CRP still needs to be investigated in large prospective studies^36^. The authors speculate that future quantification of circulating CRP before starting ART may help to identify patients with poor ART prognosis, leading to ART cycle cancellation or to preconception treatment to minimize the medical risks and costs^36^.

Leptin is an adipokine and key regulator of food intake and energy homeostasis mainly by acting on the central nervous system^37^ and circulating levels of leptin reflect body fat stores^38^. Leptin also affects the hypothalamic–pituitary–gonadal axis and the secretion of gonadotropins. Thus, leptin plays a central role in reproduction and may be one component explaining the negative effect of obesity on IVF outcome^39^. Interestingly, in women undergoing IVF, lower levels of leptin in follicular fluid were associated with a higher probability of live birth, suggesting a direct effect of leptin on ovarian function^40^. Also, based on in vitro data, it has been shown that leptin inhibits granulosa cell-produced anti-Müllerian hormone^41^.

Adipocyte fatty acid-binding protein (AFABP) is another adipokine produced mainly in adipocytes, but also in macrophages to some extent. It acts as a chaperone for free fatty acids but has also been identified as a signaling molecule^42^. Increased circulating concentrations of AFABP are seen in obesity and have been associated with development of insulin resistance and cardiovascular disease^43^. It has been speculated that AFABP is not only a biomarker of the metabolic syndrome in the steady state, but also a marker of weight change in dynamic situations^44^. This may explain why delta AFABP was negatively correlated with delta leptin, in women from the original intervention group, in the present study. This negative relationship indicates that metabolic steady state may not have been completely reached in this group of women after their weight loss. The potential role of AFABP in fertility is not known but it has been suggested to affect uterine receptivity and the establishment and maintenance of pregnancy^45^.

BMI is the widely used measure to evaluate obesity but other techniques or measures must be used to differentiate lean from fat mass and mass distribution. Large variations in body composition may be observed in subjects within the same BMI category. In a recent study of body fat distribution and ART outcomes in 788 women scheduled for IVF, it was reported that the body fat distribution, especially measured in terms of waist circumference, was more relevant than BMI to predict pregnancy rates^46^. Therefore, although not confirmed here, the abdominal adipose tissue, including the visceral depot, may be of particular interest in future mechanistical studies^47^.

The link between obesity and reduced insulin sensitivity (as for example measured by HOMA; homesostasis model assessment index) is well established^48^, and a majority of studies in the field also report that circulating FFA are elevated in obesity^49^. Both insulin resistance and FFA are linked to inflammation^48^. Thus, although not included as variables in the current study, both HOMA and FFA are adipose tissue-related markers with potential impact on pregnancy and live birth outcome of IVF in overweight and obese women. In a recent retrospective cohort study of women undergoing IVF, HOMA was not associated with clinical pregnancy, live birth, and miscarriage^50^. Others report that insulin resistant women with PCOS have a lower implantation rate than non-insulin resistant women with PCOS, controlled for confounding factors including age, BMI, free androgen index and lipid profiles^51^. Insulin resistance has been reported to increase the risk of spontaneous abortion after IVF^52^. Further, a previous study with focus on serum levels of a number of specific FFAs reported that elevated serum α-linolenic acid is associated with decreased embryo implantation rate and chance of pregnancy in women undergoing IVF while total FFA is not^53^. Clearly, future work is needed to further evaluate the role of insulin resistance and circulating total and specific FFAs in reproductive processes, and as predictive factors of pregnancy or live birth rate after IVF, in women of varying BMI.

The strength of this post-hoc analysis is that samples and data are extracted from a large RCT and that all included samples fulfilled standardizing criteria^15^. The live birth rate (24.6%) in the group of women included in this post-hoc analysis was similar to the live birth rates in the RCT (29.6% and 27.5%, intervention and control group, respectively). These results also correspond to information from the National Swedish Quality Registry for this time period (28.0% live birth/embryo transfer)^54^. A limitation is the relatively small number of women which limited our power and the number of independent variables allowed in the regression analysis. In addition, only women with moderately elevated BMI (≥ 30 and < 35 kg/m^2^) were included in the original RCT. Women with higher BMI are not offered publicly funded IVF in the Nordic countries. Future, larger, studies will allow evaluation of the significance of other adipose tissue-related biomarkers as predictors of live birth rate after IVF. Several adipokines, and metabolic variables such as insulin sensitivity and FFA, would be interesting in this context.

## Conclusion

In the present study, neither low-grade inflammation in terms of hsCRP, other circulating inflammatory or metabolic markers released from adipose tissue (leptin, AFABP) nor anthropometric measures of adiposity or adipose tissue distribution (BMI, waist circumference, waist-to-height ratio) were identified as predictive factors of live birth after IVF in a cohort of women with a wide range of BMI indices. More research is needed to determine the causes of the influence of overweight and obesity on pregnancy and live birth rates after IVF.

## Supplementary Information

Supplementary Table 1.

## Data Availability

The datasets used and analyzed during the current study are available from the corresponding author on reasonable request.

## Abbreviations

AFABP: Adipocyte fatty acid-binding protein
ART: Assisted reproductive technique
BMI: Body mass index
CV: Coefficient of variation
ELISA: Enzyme-linked immunosorbent assay
FFA: Free fatty acids
GnRH: Gonadotropin-releasing hormone
HOMA: Homeostasis model assessment index
HPG: Hypothalamic–pituitary–gonadal
hsCRP: High-sensitivity C-reactive protein
ICSI: Intracytoplasmic sperm injection
IVF: In vitro fertilization
LCD: Low calorie diet
PCOS: Polycystic ovary syndrome
RCT: Randomized controlled trial
SHBG: Sex hormone-binding globulin
SD: Standard deviation
WHtR: Waist-to-height ratio
