# Supplementary material for: Inflammatory and metabolic markers in relation to outcome of in vitro fertilization in a cohort of predominantly overweight and obese women
Source: Sci Rep. 2022 Aug 3;12:13331. doi: 10.1038/s41598-022-17612-2 (PMC9349206; doi:10.1038/s41598-022-17612-2)
Supplement: Supplementary file 1 — Supplementary Table 1. [file 41598_2022_17612_MOESM1_ESM.docx]

**ADDITIONAL DATA**

**Supplemental table 1.** Characteristics of the cohort at the examination closest to IVF (n=187).

|  | *Mean ± SD* | *Range* |
| --- | --- | --- |
| P-Glucose (mmol/L) | 5.0 ± 0.54 | 3.9–7.5 |
| B-Hb (g/L) | 137.2 ± 8.98 | 112.0–170.8 |
| S-Cholesterol (mmol/L) | 4.8 ± 0.90 | 2.8–7.7 |
| S-Triglycerides (mmol/L) | 1.1 ± 0.51 | 0.4–2.9 |
| S-HDL (mmol/L) | 1.4 ± 0.34 | 0.8–2.4 |
| S-LDL (mmol/L) | 3.1 ± 0.79 | 1.3–5.4 |
| S-TSH (mIE/L) | 2.0 ± 1.13 | 0.01–7.2 |
| S-fT4 (nmol/L) | 15.7 ± 2.70 | 9.6–28.8 |
| S-ASAT (μkat/L) | 0.4 ± 0.14 | 0.2–1.3 |
| S-ALAT (μkat/L) | 0.4 ± 0.34 | 0.1–3.0 |
| S-SHBG (nmol/L) | 48.0 ± 23.85 | 3.3–171.0 |
| S-Testosterone (nmol/L) | 1.4 ± 0.59 | 0.01–3.7 |
| S-Estradiol (pmol/L) | 318.1 ± 316.29 | 0.04–1970.0 |
| PCOS (%, (n)) | 27.3 (51) |  |
| Smoking (%, (n)) | 8.0 (15) |  |
